# Collectanea Medica: Consisting of Anecdotes, Facts, Extracts, Illustrations, &c.

**Published:** 1823-10

**Authors:** 


### COLLECTANEA MEDICA:

CONSISTING OF

ANECDOTES, FACTS, EXTRACTS, ILLUSTRATIONS, &c.

*Relating to the History or the Art of Medicine, and the Collateral Sciences.*

---

Floriferis, ut apes, in saltibus omnia libant,
Omnia nos, itidem, depascimur aurea dicta.

---

ART. I.—*Extracts from "Medical Jurisprudence."* By J. A. PARIS, M.D. &c. and J. S. M. FONBLANQUE, Esq.

### *Medical and Physiological Illustrations of Insanity.*

As the duties of the jurist and physiologist in the investigation of mental derangement are distinct in their nature, if not different in their object, so shall we find that the abstract terms used to denote the form or degree of the malady have received from the two professions a somewhat different latitude of acceptation. For legal purposes, the adoption of the term "*non compos mentis*," from the amplitude of its construction, gets rid of those nicer distinctions and difficulties which the pathologist is bound to encounter and investigate. The lawyer only inquires whether such a state of mind exists as actually disqualifies the person in question from conducting himself with propriety, or managing his affairs; but the medical evidence is bound not only to give his opinion upon the case, but to state the reasons which may have influenced his decision; and hence the necessity of his becoming practically acquainted with those physiological distinctions to which we have alluded. It has been stated that there are two conditions of the human mind, either of which very justly deprives the subject of the control of his person and property, and takes away from him all criminal responsibility,—viz. Idiotcy, *(amentia,)* or a total deficiency of intellectual power; and Madness, or a morbid perversion of it. Between these two states we shall not have much difficulty in discriminating: the idiot cannot reason at all; the madman reasons falsely: the idiot acts from animal appetency, he has no will; the madman wills, but, his reason being disturbed, his actions are not compatible with the usual relations of society.

Idiotcy may exist from birth, *(amentia congenita,* Cull. Syn. lxv. 1;) or it may be the effect of old age, *dotage, (amentia senilis,* Cull. Syn. lxv. 2;) or it may arise at any period of life from the operation of various causes affecting the functions of the brain,—such as epileptic fits, intense study, intemperance, the depressing passions, especially grief, fevers, paralysis, and mania, *(amentia acquisita,* Cull. Syn. lxv. 3.) In some cases fatuity is symptomatic of another disease.

The law, as we have already stated, makes an important distinction between that species of idiotcy which is congenital, *de nativitate*, and that which may occur in after life; and upon this point, as well as upon the extent of the malady, and the probability of its cure, the medical practitioner may be called upon to give an opinion. In cases of congenital idiotcy, there will not be much difficulty in pronouncing judgment; for, as it arises from malformation of the cerebral organ, the prognosis must be adverse to every hope of recovery; while the characteristic physiognomy of the unfortunate individual is generally so striking as to enable the common observer at once to ascertain the existence of idiotcy. The vague expression of his countenance is commonly associated with an awkwardness in the gait, which would seem to depend upon a defect in the muscular powers: there is, moreover, a degree of incontinence with respect to the excretory discharges of his body; and, owing to a carelessness in not swallowing the saliva, there is a constant drivelling from the mouth. The speech is imperfect; and the extent of this deficiency may, in general, be considered as a good indication of the degree of fatuity, for it is necessary to state that all idiots are not of the same degree of intellectual depravity. Some possess more memory than others, and display a talent for imitation; they will whistle tunes correctly, and repeat passages from books which they have been taught by ear, but they are incapable of comprehending what they repeat. Under such circumstances, medical evidence may be required for the purpose of obtaining an estimate of the capacity of such an individual, and upon this subject Dr. Haslam has offered the following judicious remarks: "It has occurred to me, in many instances, to be consulted concerning persons whose minds have been naturally weak, or enfeebled by disease; and it always appeared that, by patient inquiry, a satisfactory estimate of their capacity might be instituted. The person exercising his judgment upon this question ought particularly to ascertain the power of the idiot's attention; since his knowledge of objects, and his memory of them, will depend on the duration of his attention. It will also be indispensably necessary to investigate his comprehension of numbers, without which the nature of property cannot be understood: if a person were capable of enumerating progressively to the number ten, and knew the force and value of the separate units, he would be fully competent to the management of property; if he could comprehend that twice two composed four, he could find no difficulty in understanding that twice, or twain ten, constituted twenty. This numeration also presumes he comprehended that so many taken from ten, or subtracted, which is the converse, would leave so many as the remainder. Without such capacity, no man, in my own opinion, could understand the nature of property, which is represented by numbers of pounds, shillings, and pence. The same imbecility of mind is often produced in adults, and in those of advanced age, by paralytic or epileptic attacks, and from various affections of the brain, and requires the same accurate investigation to determine on the competency of such persons to be entrusted with the management of themselves and affairs."

In cases of *amentia acquisita*, our prognosis must be directed by different circumstances: the faculties of a person may only be in abeyance, and may revert to a state of sanity, either spontaneously or from judicious treatment, or they may be only partially affected. It however deserves notice that, in extent of mortality, the most fatal of all the states of mental disorder is amentia acquisita. It has been computed that, in the French hospitals, a full moiety of the fatuous die; at the same time it appears, from the reports of lunatic asylums, that this disorder is sometimes cured.

Authors who have treated on the subject of Insanity have anxiously attempted to frame a definition of the malady; and, by compressing into a short sentence its prominent and distinguishing phenomena, to establish a fixed and essential character. In this attempt each author has fundamentally differed, and to enumerate their plans would be only to expose their failures. The truth is, that the varied and mutable phenomena of insanity will ever mock the grasp of the nosologist: instead, therefore, of endeavouring to discover an infallible definition, it will be of much greater importance to investigate the circumstances which should guide the medical witness in a decision that may annul a man's dominion over property, involve his contracts and other acts, which otherwise would be binding, and take away his responsibility for crimes. Modern authors, according to the system of the Grecian writers, have generally divided mental derangement into two classes,—*mania* and *melancholia;* the former being distinguished by a state of extraordinary excitement, the latter by great depression, although they are frequently convertible affections.

Mania may be said to be a state of mental alienation, accompanied by an unusual ferocity in language and deportment, and by a comparative insensibility to ordinary stimuli.

Melancholia is a form of insanity which is always attended with some seemingly groundless, but very anxious fear, by which the person is plunged into a gloomy and desponding state, that not unfrequently leads to the commission of suicide.

The approaches of insanity have been as variously described by different authors, as the characters by which the malady itself is to be distinguished: indeed, the precursory symptoms of mania are extremely indefinite and variable. Dr. Haslam observes, that "the attack is almost imperceptible; some months usually elapse before it becomes the subject of particular notice, and fond relatives are frequently deceived by the hope that it is only an abatement of excessive vivacity, conducing to a prudent reserve and steadiness of character. A degree of apparent thoughtfulness and inactivity precedes, together with a diminution of the ordinary curiosity concerning that which is passing before them; and they therefore neglect those objects and pursuits which formerly proved sources of delight and instruction; the sensibility appears to be considerably blunted; they do not bear the same affection towards their parents and relations; they become unfeeling to kindness, and careless of reproof; if they read a book, they are unable to give any account of its contents; sometimes, with stedfast eyes, they will dwell for an hour on one page, and then turn over a number in a few minutes. Their sleep is disturbed, and they awake in the morning in a state of great disquietude and anxiety. As the malady becomes further developed, the symptoms are less equivocal: the unhappy objects become loquacious and disposed to harangue, and decide promptly and positively upon every subject that may be started; soon after, they are divested of all restraint in the declaration of their opinions of those with whom they are acquainted; their friendships are expressed with fervency and extravagance, their enmities with intolerance and disgust. They now become impatient of contradiction, and scorn reproof; for supposed injuries, they are inclined to quarrel and fight with those about them: at length suspicion creeps upon the mind, they are aware of plots which had never been contrived, and detect motives that were never entertained."

This picture, however, must be only regarded as displaying the ordinary occurrences which precede the attack; its approaches are sometimes distinguished by a very different train of symptoms. The late Dr. John Monro has remarked that "high spirits, as they are generally termed, are the first symptoms of this kind of disorder: these excite a man to take a larger quantity of wine than usual; and the person thus affected, from being abstemious, reserved, and modest, shall become quite the contrary, drink freely, talk boldly, obscenely, swear, sit up till midnight, sleep little, rise suddenly from bed, go out a hunting, return again immediately, set all his servants to work, and employ five times the number that is necessary: in short, every thing he says or does betrays the most violent agitation of mind, which it is not in his power to correct; and yet, in the midst of all this hurry, he will not misplace one word, or give the least reason for any one to think he imagines things to exist that really do not, or that they appear to him different from what they do to other people. They who see him but seldom admire his vivacity, are pleased with his sallies of wit, and the sagacity of his remarks: nay, his own family are with difficulty persuaded to take proper care of him, until it becomes absolutely necessary, from the apparent ruin of his health and fortune."

The patient under the influence of the depressing passions will exhibit a train of symptoms altogether different: the countenance wears an anxious and gloomy aspect; he is little disposed to speak; he retires from the company of those with whom he formerly associated, secludes himself in obscure places, or lies in bed the greater part of his time; frequently he will keep his eyes fixed on some object for hours together, or continue them an equal time "bent on vacuity." He next becomes fearful, and conceives a thousand fancies; often recurs to some immoral act which he has committed, or imagines himself guilty of crimes which he never perpetrated; believes that God has abandoned him, and with trembling awaits his punishment. Frequently he becomes desperate, and endeavours by his own hands to terminate an existence which appears to be an afflicting and hateful incumbrance.

The approaches of insanity are, however, not always slow and progressive: the unhappy victim is sometimes seized without any warning, and, where crimes have been perpetrated under such circumstances, it becomes extremely embarrassing both to the judgment of the physician and to the decision of the court. Each case, however, must rest upon its own particular merits, duly to be weighed and considered both by the judge and jury, lest, to use the expressions of Sir Matthew

Hale, "there be on the one side a kind of inhumanity towards the defects of human nature; or, on the other side, too great an indulgence given to great crimes."

It has been very justly observed, that, to constitute insanity, it is not necessary to exhibit the ferocity of a wild beast, nor to perform the antics of a buffoon: the most ordinary observer can tell when a person is furiously mad; but, in many cases, "such thin partitions do the bounds divide," that all the skill and discernment of a medical practitioner is required to establish the fact of insanity. It is to such cases as are more likely to become subjects of legal investigation, that the following observations particularly apply. Sir Matthew Hale says, "there is a *partial* insanity and a *total* insanity: the former is either in respect to things, *quoad hoc vel illud insanine*, where persons are perfectly rational, except on some one particular subject." This fact is universally admitted, constituting a form of mental alienation to which M. Esquirol has bestowed the name of *monomania*, and of which every work on insanity abounds with examples. It is in such cases that the value of medical sagacity and experience becomes apparent; and the full development of the real state of the patient's mind and opinions will, in some instances, require considerable time and patience. "It is nearly impossible," says Dr. Haslam, "to give any specific directions for conducting such an examination as shall inevitably disclose the delusions existing in the mind of a crafty lunatic; but in my own opinion it is always to be accomplished, provided sufficient time be allowed, and the examiner be not interrupted. It is not to be effected by directly selecting the subjects of his delusion; for he will immediately perceive the drift of such inquiries, and endeavour to evade, or pretend to disown them: the purpose is more effectually answered by leading him to the origin of his distemper, and tracing down the consecutive series of his actions and association of ideas: in going over the road where he has stumbled, he will infallibly trip again." There is, says Dr. Male, a madness which shows itself in words, and another in actions: a lunatic may be coherent in conversation, but insane in conduct; he may be rational when under the restraint of a mad-house, but, when released, and at liberty to act according to the impulse of his hallucination, will shew by his conduct that he is really insane.

The medical practitioner, in delivering an opinion that may involve the liberty of the person, cannot well be too guarded in his evidence. As each case must rest upon its own merits, the subject scarcely admits of any general elucidation beyond that which we have already endeavoured to bestow, and the plan of our work must of necessity preclude the more minute details. We must, however, here observe, that coercion should never be employed but as a protecting restraint, to guard the patient from doing mischief to himself, or offering violence to others; and for this purpose the straight-waistcoat is generally sufficient. Formerly, coercion was employed with a degree of severity that amounted to vindictive punishment; recourse was even had to the whip, and stripes were actually inflicted by medical direction; while asylums for the reception of the insane were considered as prisons for safe custody and punishment, rather than as hospitals for the treatment and cure of this most dreadful malady.

*Whether there has been any lucid interval, and of what duration?*

This is a question which a medical witness is always called upon to answer. By the term *lucid interval*, we are not to understand a *remission* of the malady, but a total suspension of it,—a complete, although only a temporary, restoration of reason. The question is generally beset with difficulties, and requires all the penetration and experience of the physician to arrive at a safe conclusion; for, in many cases, the patient is enabled for a limited period to converse rationally, and, where he is desirous of carrying any particular plan into execution, to dissemble so completely as to impose with success upon his attendants: of which the following case, related by Dr. Haslam, may serve as an excellent illustration. "A lunatic having received, or fancied he had received, an injury from his keeper, at the lunatic asylum at Manchester, threatened to be revenged, for which he was punished by confinement. He was afterwards a patient in Bethlem Hospital, and gave Dr. Haslam an account of the transaction, of which the following is an abbreviation. 'Not liking this situation, I was induced to play the hypocrite: I pretended extreme sorrow for having threatened him, and, by an affectation of repentance, induced him to release me. For several days I paid him great attention, and lent him every assistance: he seemed much pleased with the flattery, and became very friendly in his behaviour towards me. Going one day into the kitchen, where his wife was busied, I saw a knife: this was too great a temptation to be resisted; I concealed it, and carried it about with me. For some time afterwards the same friendly intercourse was maintained between us; but, as he was one day unlocking his garden-door, I seized the opportunity, and plunged this knife, up to the hilt, in his back.'" There is a species of insanity which has been called *intermittent*, in which the patient is perfectly rational for a considerable interval. The malady often recurs two or three times in a year, and lasts several weeks, the subject of the hallucination being always the same.

*Whether there is a probable chance of recovery; and, in case of convalescence, whether the cure is likely to be permanent?*

The prognosis, or means of ascertaining the probable event of mental derangement, is founded on the consideration of many different circumstances,—such as the particular modification of the malady; the violence of the symptoms; the duration and frequency of the attack; its causes; the age, sex, constitutional temperament, and hereditary dispositions, of the affected individual; the general state of his health; and the particular nature of his bodily maladies: upon each of which we shall offer a few observations. It has been remarked, that those affected with furious mania recover in a larger proportion than those who suffer under the depressing influence of melancholy; but that, when the maniacal and melancholic states alternate, the hope of recovery is farther diminished. The probability of cure is also more or less according to the duration of the disease. When, however, it has acquired a systematic character, it becomes very difficult to remove it; so that, after it has continued upwards of a year, patients at public asylums, as in Bethlem and St. Luke's, are pronounced incurable, and treated accordingly. In considering the causes of mania, we must class them in two divisions—*predisposing* and *exciting*. Among the former of these causes stand hereditary predisposition; injuries of the brain, (these also belong to the class of exciting causes;) certain bodily diseases; and a peculiar temperament. Among the latter we may first enumerate those of a *physical* nature,—as frequent intoxication; fever; mercurial medicines, largely administered; the suppression of periodical or occasional discharges and secretions; parturition; injuries to the head from external violence, &c. The *moral* causes include those emotions which are conceived to originate from the mind itself, and which, from their excess, tend to distort the natural feelings; or, from their repeated accessions, and unrestrained indulgence, at length overthrow the barriers of reason and established opinion: such are the gusts of violent passion, and the protracted indulgence of grief; the terror impressed by erroneous views of religion; the degradation of pride; disappointment in love; and sudden fright.

Of hereditary disposition we may observe, that there does not appear to be any malady more obviously dependent upon its influence than that of madness; for, even if one generation escape, the taint is presumed to cling to the succeeding branches until, either by admixture with a purer stock, or by education or management, it is neutralized or drained away. In forming a prognosis, it therefore becomes the first object of inquiry, whether any branch of the patient's family has ever manifested any symptoms of the disease? for, where this is made out, our expectations of permanent recovery must be slender; and, even should the patient become convalescent, he will be liable to a relapse from every fresh exposure to the exciting causes. Injuries about the head may be considered as both the predisposing and exciting causes of insanity; for a fracture of the cranium has been known to produce disorder in persons who had never betrayed the least obliquity previous to the accident, and whose families had never manifested the slightest disposition to the malady. Although mental derangement has been observed in persons of every habit and temperament, yet there is certainly a complexion which may be said to predominate in these cases: Dr. Haslam, for instance, has stated that, out of 265 patients in Bethlem Hospital, 205 were found to be of a swarthy complexion, with dark or black hair; the remaining sixty having a fair skin, and light-brown or red hair. Among the most powerful exciting causes of derangement of intellect in those predisposed to the malady, are to be classed the moral causes which produce mental distress and uneasiness. At the eventful era of the French revolution, and for some years after, the lunatic establishments of France were inundated by its victims; and Dr. Burrows observes, that the annals of insanity will satisfactorily show that there never was, in any country, a sudden increment of insane persons, without some powerful and evident excitation, physical, moral, theological, or political. I have, says Zimmerman, had occasion to see all the great hospitals in Paris, and have distinguished in them three kinds of maniacs: the men who had become so through pride, the girls through love, and the women through jealousy.

The use of ardent spirits or wine, to a person predisposed to insanity, is always dangerous: under the same circumstances, a long course of mercurial remedies has been found mischievous. The suppression of accustomed evacuations is also a frequent cause of mania, and the restoration of them not unfrequently removes the mental affection. Where there is in women a hereditary disposition to mania, it is frequently called into action immediately after parturition: in such cases the prognosis is favourable.* On the other hand, it has been remarked that, in our climate, women are more frequently affected with insanity than men; and it has been considered very unfavourable to recovery if they should be worse at the period of menstruation, or have their catamenia in very small or immoderate quantities. We have already noticed local injuries of the head among the predisposing causes; we may also observe, in this place, that they not unfrequently prove an exciting one: in the case of Hadfield, the insanity was occasioned by a blow on the skull.

---

* During ten years, eighty patients of this description were admitted into Bethlem Hospital, fifty of whom perfectly recovered.

